# Does periodontal treatment improve rheumatoid arthritis disease activity? A systematic review

**DOI:** 10.1093/rap/rkac061

**Published:** 2022-08-17

**Authors:** Zhain Mustufvi, Joshua Twigg, Joel Kerry, James Chesterman, Sue Pavitt, Aradhna Tugnait, Kulveer Mankia

**Affiliations:** School of Dentistry, University of Leeds; School of Dentistry, University of Leeds; Library and Information Service, Leeds Teaching Hospitals NHS Trust; Leeds Teaching Hospitals NHS Trust; School of Dentistry, University of Leeds; School of Dentistry, University of Leeds; Leeds Institute of Rheumatic and Musculoskeletal Medicine, University of Leeds, Leeds, UK

**Keywords:** periodontal disease, RA, periodontal treatment, disease activity, non-pharmacological

## Abstract

**Objectives:**

The association of periodontal disease in people diagnosed with RA is emerging as an important driver of the RA autoimmune response. Screening for and treating periodontal disease might benefit people with RA. We performed a systematic literature review to investigate the effect of periodontal treatment on RA disease activity.

**Methods:**

Medline/PubMed, Embase and Cochrane databases were searched. Studies investigating the effect of periodontal treatment on various RA disease activity measures were included. The quality of included studies was assessed. Data were grouped and analysed according to RA disease outcome measures, and a narrative synthesis was performed.

**Results:**

We identified a total of 21 studies, of which 11 were of non-randomized experimental design trials and 10 were randomized controlled trials. The quality of the studies ranged from low to serious/critical levels of bias. RA DAS-28 was the primary outcome for most studies. A total of 9 out of 17 studies reported a significant intra-group change in DAS-28. Three studies demonstrated a significant intra-group improvement in ACPA level after non-surgical periodontal treatment. Other RA biomarkers showed high levels of variability at baseline and after periodontal treatment.

**Conclusion:**

There is some evidence to suggest that periodontal treatment improves RA disease activity in the short term, as measured by DAS-28. Further high-quality studies with longer durations of follow-up are needed. The selection of the study population, periodontal interventions, biomarkers and outcome measures should all be considered when designing future studies. There is a need for well-balanced subject groups with prespecified disease characteristics.

Key messages

- A short course of periodontal treatment can significantly improve RA disease activity.
- Periodontal treatment might influence serum ACPA levels in people with RA and co-existent periodontitis.
- Further high-quality intervention studies with longer study durations are needed.

## Introduction

RA is an autoimmune inflammatory condition that primarily affects the joints. RA autoimmunity is thought to be initiated at mucosal sites, such as the oral cavity, lung and gastrointestinal tract. At these sites, local inflammation [e.g. periodontitis (PD)] can be driven by genetic or environmental risk factors (e.g. cigarette smoke). The combination of mucosal inflammation and local bacterial dysbiosis might be responsible for triggering the RA autoimmune response, in particular ACPA, the serological hallmark of RA [1]. Of the mucosal sites, the periodontium has been particularly well studied, perhaps owing to the relative ease of accessibility for assessment and sampling, in addition to the putative link between specific oral bacteria and RA [2].

Periodontitis is an inflammatory condition of the tooth supporting structures initiated by microbial biofilms on the tooth surface (dental plaque) and exacerbated by a dysregulated host response. There is also a similar dysregulation of the pro-inflammatory cytokines to that seen in RA [3]. The resulting inflammation leads to destruction of the periodontium, which can lead to tooth loss. The gold standard for treatment is patient education/motivation and non-surgical periodontal treatment (NSPT), involving scaling of the teeth to disrupt bacterial biofilms.

There is mounting evidence to support a relationship between RA and PD. It has been found that periodontal disease is more prevalent in people with RA [4]. PD is also more prevalent in ACPA^+^ at-risk individuals without arthritis [5]. A key periodontal pathogen, *Porphyromonas gingivalis*, has a peptidylarginine deiminase enzyme that can citrullinate cytoskeletal filaments, which might promote the production of ACPAs. It is therefore hypothesized that changes in the oral microbiome in PD could be a trigger for RA-related autoimmunity. A study investigating microbial composition of subgingival plaque in CCP^+^ subjects at risk of RA found dysbiotic subgingival microbiome composition in addition to an increased prevalence of *P. gingivalis* compared with healthy controls [6].

Building on the putative association between PD and RA, a logical question is: can periodontal therapy influence disease activity and disease progression in RA? Recent reviews have found improved DASs after non-surgical periodontal treatment [7–9]. We aim to add to the evidence by providing an up-to-date review and scrutinize, in detail, the effect of periodontal treatment on the various RA disease activity measures.

## Methods

### Search strategy

A systematic literature search was designed with input from an expert librarian and informatician at Leeds Teaching Hospitals NHS Trust using a combination of key words and MESH terms. The search was conducted according to a prespecified protocol and the Preferred Reporting Items for Systematic Reviews and Meta-Analysis (PRISMA) guidelines [10]. Searches were performed on Medline/PubMed (from 1944 to January 2022), OVID Embase (from 1944 to January 2022), Cochrane Central Register databases (from 1944 to January 2022). A hand search was performed through the references of the articles found to identify any further papers. Detailed search terms are listed in Supplementary Data S1, available at *Rheumatology Advances in Practice* online. Table 1 details the inclusion/exclusion criteria.

**Table 1:** Inclusion/exclusion criteria

Inclusion criteria	Exclusion criteria
RA was defined according to internationally accepted criteria	Non-relevant study populations
Periodontal disease was defined according to internationally accepted criteria	Non-intervention studies
Study population had a clinically acceptable periodontal intervention as part of the trial	Studies with incomplete follow-up or missing data
Study population had a minimum follow-up period of 4 weeks	Studies not reporting on relevant RA outcome measures
Baseline and follow-up data included periodontal and RA parameters	Studies including not clinically acceptable periodontal treatment
Relevant RA outcome measures were recorded, including DAS-28, ACPA, ESR, CRP, RF, early morning stiffness, HAQ and other ancillary biomarkers	Unclear methodology
Studies had data that could be extracted	

### Data extraction

Screening of search results was undertaken independently by two authors (Z.M. and J.T.). Any disagreement was resolved through discussion and, where necessary, arbitration by a third author (K.M.).

For each article, the effect of NSPT on RA disease activity was analysed, and the following themes emerged from the data: the effect of treatment on DAS-28, ACPA, ESR and CRP, RF, swollen and tender joints, morning stiffness, HAQ and other ancillary RA biomarkers.

Effect measures of NSPT on outcome measure were calculated as the mean difference between baseline and follow-up (intra-group comparison) and/or the mean difference between groups (inter-group comparison). Where possible, the s.e.m. was provided. Data were tabulated for a narrative synthesis and grouped by RA disease outcome measure.

### Quality assessment

Included articles were quality appraised using the Risk Of Bias In Non-randomized Studies—of Interventions (ROBINS-I) [11] and the Cochrane risk of bias for randomized trials (v.2 [12]) tools, according to study design. Risk of bias was undertaken independently by two authors (Z.M. and J.T.), with disagreement resolved through discussion and, where necessary, arbitration from a third author (K.M.).

### Registration

The systematic review was not registered. There was no deviation from the protocol throughout the review.

## Results

**Fig. 1:**
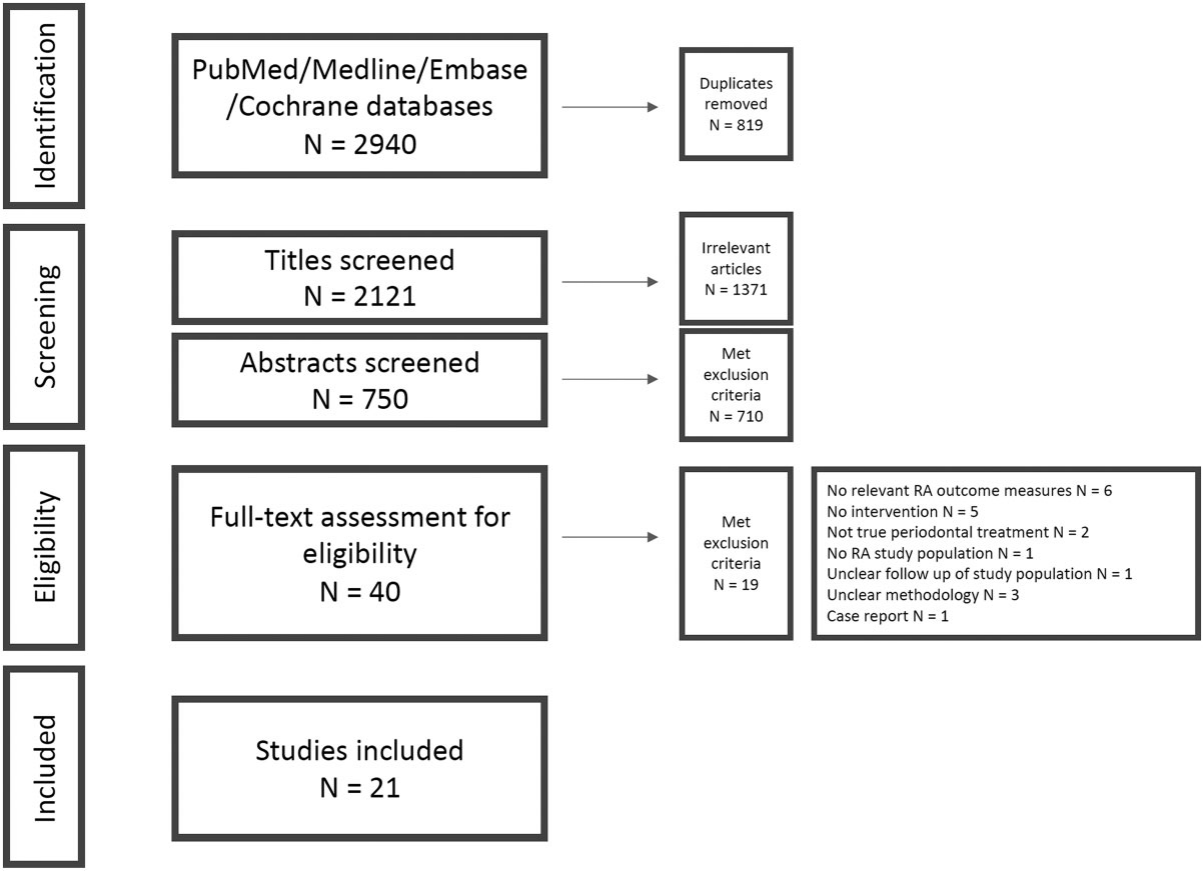
PRISMA flow diagram detailing the systematic search

The screening results are summarized in the PRISMA flow diagram (Fig. 1). Supplementary Table S1, available at *Rheumatology Advances in Practice* online, details the excluded studies. A total of 21 articles were included in the analysis; 10 were of randomized controlled trial design and 11 of non-randomized intervention design. The study characteristics are detailed in Table 2.

**Table 2:** Characteristics of included studies

Author(s) (year)	Study population and intervention arms	Baseline RA disease duration and severity	RA treatment of study population	Study design	Study duration	RA disease activity measures	Key outcome
Al-Katma *et al.* (2007) [13]	*n* = 29 subjects with RA + PD (1) 17 OHI + NSPT (2) 12 no treatment	RA duration not reported Moderate disease activity in both arms	Not reported	Single-centre, two-arm, parallel-group, randomized controlled trial	8 weeks	DAS-28-ESR ESR SJ, TJ Morning stiffness VAS	DAS-28 mean reduction of 0.6 (0.5), *P* < 0.05 in group 1 (OHI + SRP) compared with group 2 (no treatment)
Ortiz *et al.* (2009) [14]	*n* = 40 (A) 10 subjects with RA + PD who had NSPT (B) 10 subjects with RA + PD: no treatment (C) 10 subjects with RA + PD who had NSPT and took anti-TNF medication (D) 10 subjects with RA + PD: no treatment but took anti-TNF medication	RA duration not reported RA severity: Group A: 100% severe Group B: 70% severe, 30% moderate Group C: 80% severe, 20% moderate Group D; 50% severe, 50% moderate	50% csDMARDs prerandomization into treatment/non-treatment arms 50% csDMARDs + anti-TNF prerandomization into treatment/non- treatment arms	Single-centre, four-arm, parallel-group, randomized controlled trial	8 weeks	DAS-28-ESR ESR SJ, TJ VAS	Group A: DAS-28 mean reduction of 1.58 (0.46), *P* < 0.01, at 6 weeks *vs* baseline Group C: DAS-28 mean reduction 1.42 (0.46), *P* < 0.05, at 6 weeks *vs* baseline Statistically significant difference in DAS-28 between treatment arms and non-treatment arms (*P* = 0.005)
Pinho *et al.* (2009) [15]	*n* = 75 (1) 15 subjects with RA + PD, NSPT (2) 15 subjects with RA + PD, no treatment (3) 15 subjects with PD, NSPT (4) 15 edentulous subjects, no treatment (5) 15 healthy controls	RA duration 6 months to 10 years RA severity: low disease activity in treatment group	Not reported	Single-centre, five-arm, parallel-group, non-randomized intervention study	6 months	DAS-28-ESR ESR CRP AAG HAQ	Group 1: DAS-28 mean reduction of 0.65 (0.37) at 3 months, *P* < 0.05 No statistically significant reduction at 6 months
Erciyas *et al.* (2012) [16]	*n* = 60 (1) 30 RA subjects with low disease activity who had NSPT (2) 30 RA subjects with moderate/high disease activity who had NSPT	RA duration: low disease group 7.1 years (3.9), moderate/high disease group 7.4 (5.1) 50% low disease activity, 50% moderate/high disease activity	Low disease activity 23.3% anti-TNF, 93.3% csDMARDs, 73.3% CSs Moderate/high disease activity: 16.7% anti-TNF, cs90% DMARDs, 86.7% CSs	Single-centre, two-arm, parallel-group, non-randomized intervention study	3 months	DAS-28* CRP ESR TNF-α	Low disease activity group: DAS-28 mean reduction 0.24 (0.12), *P* < 0.01 Moderate/high disease activity group: DAS-28 mean reduction 2.31 (0.21), *P* < 0.01
Bıyıkoğlu *et al.* (2013) [17]	*n* = 30 (1) 15 subjects with RA + PD and NSPT (2) 15 subjects with PD and NSPT	RA duration 6.40 (4.46) years 26.6% low 66.7% moderate 6.7% high	100% csDMARDs 6.7% csDMARD + anti-TNF 93.3% prednisolone	Single-centre, two-arm, parallel-group, non-randomized intervention study	6 months	DAS-28* CRP ESR RF GCF and serum IL-1β TNF-α	In group 1: DAS-28 mean reduction of 2.01 (0.31) at 1 month, *P* < 0.01; DAS-28 at 3 and 6 months not significantly different
Okada *et al.* (2013) [18]	*n* = 55 (1) 26 subjects with RA + PD, NSPT (2) 29 subjects with RA + PD, no treatment	RA duration: treatment group 12.2 (2.5) years; control group 12.9 (2.3) years Treatment group 65.4% remission, 26.9% low, 7.7% medium Control group 62.1% remission, 24.1% low, 13.8% moderate	Not reported	Single-centre, two-arm, parallel-group, randomized controlled trial	8 weeks	DAS-28-CRP SJ, TJ CRP ACPA RF IL-6 TNF-α IgG (*Porphyromonas gingivalis* ) Amino acid volume Citrulline	Group 1: DAS-28 mean reduction of 0.37 (0.04) Group 2: no reduction in DAS-28 Inter-group comparative significance: *P* = 0.02
Roman-Torres *et al.* (2015) [19]	*n* = 24 (1) 12 subjects with RA + PD, NSPT (2) 12 subjects with PD, NSPT	RA duration 10 years RA disease activity not specified	Not reported	Single-centre, two-arm, parallel-group, non-randomized intervention study	3 months	CRP ESR	No statistically significant reduction in CRP or ESR in either group
Kurgan *et al.* (2016) [20]	*n* = 66 (1) 13 healthy controls, OHI only (2) 13 systemically healthy subjects with gingivitis, NSPT (3) 13 systemically healthy subjects, PD, NSPT (4) 14 subjects with RA + gingivitis, NSPT (5) 13 subjects with RA + PD, NSPT	RA duration: RA+PD 10 years; RA + gingivitis 7 years Low disease activity in RA + PD (median DAS-28 2.6) and RA + gingivitis (median DAS-28 2.8)	Group 4: 93% NSAIDs 50% MTX 17% SSZ 40% CSs Group 5: 77% NSAIDs 77% MTX 18% SSZ 69% CSs	Single-centre, four-arm, parallel-group, non-randomized intervention study	3 months	DAS-28* ESR CRP RF MMP-8 IL-6 PGE_2_	No reduction in DAS-28 in either group 4 or 5 Statistically significant (*P* < 0.05) reduction pre- and post-treatment in: MMP-8 in groups 3,4 and 5; PGE_2_ in groups 2 and 5; IL-6 in groups 4 and 5
Kurgan *et al.* (2017) [21]	*n* = 45 (1) 15 subjects with RA + PD, NSPT (2) 15 subjects with PD, NSPT (3) 15 healthy controls	RA duration not reported Low disease activity in group 1 (mean DAS-28 2.99)	Unclear, although biological therapy was an exclusion criterion	Single-centre, three-arm, parallel-group, non-randomized intervention study	3 months	DAS-28* ESR CRP Vessel-type plasminogen activator (t-PA) Plasminogen activator inhibitor-2	No reduction in DAS-28 Statistically significant reduction in t-PA after treatment in group 1, *P* = 0.047
Khare *et al.* (2016) [22]	*n* = 60 (1) 30 subjects with RA + PD, NSPT (2) 30 subjects with RA + PD, no treatment	RA duration not reported High disease activity in treatment and control groups (mean DAS-28 6.6 and 6.9, respectively)	Not reported	Single-centre, two-arm, parallel-group, randomized controlled trial	3 months	DAS-28* CRP ESR	In group 1, mean DAS-28 reduction of 1.05 (0.28), *P* < 0.05 Inter-group comparison shows statistically significant difference at 3 months, *P* = 0.0002
Serban (2017) [23]	*n* = 60 (1) 30 subjects with RA + PD, NSPT (2) 30 subjects with RA + PD, NSPT after study	RA duration not reported High disease activity in both arms (mean scores: group 1, 4.6; group2, 5.1)	Group 1: csDMARDs 36.6% bDMARDs 33.3% NSAIDs 10% Group 2: csDMARDs 56.5% bDMARDs 33.3% NSAIDs 20%	Single-centre, two-arm, parallel-group, randomized controlled trial	6 months	DAS-28-ESR ESR VAS TJ, SJ EuroQuol	No significant reduction in DAS-28 No significant reduction in ESR
Cosgarea *et al.* (2018) [24]	*n* = 36 (1) 18 subjects with RA + PD, NSPT (2) 18 subjects with PD, NSPT	RA duration: mean 14.88 (5.55) years Moderate disease activity (median DAS-28, 4.8)	100% csDMARDs 16.7% anti-TNF 72.2% NSAIDs 33.3% CSs	Single-centre, two-arm, parallel-group, non-randomized intervention study	6 months	DAS-28-ESR ESR CRP RF MMP-8 IL-1β IL-10	No reduction in DAS-28 in group 1 Statistically significant reduction in group 1 of CRP at 3 months *vs* baseline (*P* = 0.023) but not at 6 months *vs* baseline (*P* = 0.346)
Zhao *et al.* (2018) [25]	*n* = 64 (1) 18 subjects with PD NSPT (2) 18 subjects with RA NSPT (3) 18 subjects with RA + PD NSPT (4) 10 healthy controls NSPT	RA duration not reported Moderate disease activity in RA + PD and RA groups (mean DAS-28, 4.6 and 3.4, respectively)	Not reported	Single-centre, four-arm, parallel-group, non-randomized intervention study	4 weeks	DAS-28* ACPA CRP ESR	In group 3, there was a significant reduction in DAS-28, ACPA, CRP and ESR at 4 weeks compared with baseline. This included a reduction in DAS-28 of 1.15, *P* < 0.001
Anusha *et al.* (2019) [26]	*n* = 45 (1) 15 subjects with RA + PD, NSPT (2) 15 subjects with RA + PD, NSPT + chlorhexidine (3) 15 subjects with RA + PD, NSPT + mouthwash containing essential oils and curcumin	RA duration not reported RA disease activity not reported	Not reported	Single-centre, three-arm, triple-blinded, parallel-group, randomized controlled trial	6 weeks	ACPA ESR CRP RF	Reduction in ACPA in group 2 of 164.8 (4.82), *P* < 0.001 Reduction in ACPA in group 3 of 174.26 (4.30), *P* < 0.001
Białowas *et al.* (2019) [27]	*n* = 22 subjects with RA + PD, NSPT	RA duration not reported for subgroup Moderate disease activity (median DAS-28 4.32)	Not reported for subgroup	Single-centre before and after study of subgroup of study population	3 months	DAS-28 (ESR and CRP) CRP ESR SJ, TJ VAS SDAI CDAI HAQ Morning stiffness TNF-α MMP-3 MMP-9	Reduction in median DAS-28 (ESR) score of 0.48, *P* = 0.04 Reduction in median DAS-28 (CRP) score of 0.5, *P* = 0.002
Kaushal *et al.* (2019) [28]	*n* = 40 (1) 20 subjects with RA + PD, NSPT (2) 20 subjects with RA + PD, no treatment	RA duration not reported High disease activity in both treatment and control groups (mean SDAI, 30.53 and 28.94, respectively)	Not reported	Single-centre, two-arm, parallel-group, non-randomized controlled trial	8 weeks	ACPA CRP RF SDAI	Reduction in SDAI score of 11.51 in group 1, *P* < 0.001
Monsarrat *et al.* (2019) [29]	*n* = 22 (1) 11 subjects with RA + PD, NSPT (2) 11 subjects with RA + PD, no treatment	RA duration: treatment group 12.1 (6.5) years; control group 11.4 (8.6) years Moderate disease activity in treatment and control groups (mean, 4.24 and 3.82, respectively)	Treatment group: 73% csDMARDs 64% bDMARDs 55% glucocorticoids 36% NSAIDs Control group: 82% csDMARDs 91% bDMARDs 45% glucocorticoids 36% NSAIDs	Two-centre, two-arm, parallel-group, randomized controlled trial	3 months	DAS-28-ESR ESR CRP VAS	No statistically significant reduction in DAS-28 in either arm
Moura *et al.* (2020) [30]	*n* = 107 (1) 24 subjects with RA + PD, NSPT (2) 30 subjects with PD, NSPT (3) 23 subjects with RA, no treatment (4) 30 healthy controls	RA duration not reported. Moderate disease activity reported in both RA + PD and RA groups (mean, 4.34 and 3.69, respectively)	Not reported	Single-centre, four-arm, parallel-group, randomized controlled trial	45 days	DAS-28* ESR CRP	Reduction in DAS-28 of 1.34 (0.21), *P* = 0.011 in group 1
Elsadek & Farahat (2021) [31]	*n* = 50 (1) 25 subjects with RA + PD, NSPT + photodynamic therapy (2) 25 subjects with RA + PD, NSPT	RA duration not reported Moderate disease activity in both arms (mean group 1, 3.55; group 2, 3.68)	Group 1: NSAIDs 56% IL-6 antagonist 32% LEF 25% Group 2: NSAIDs 76% IL-6 antagonist 12% LEF 8%	Single-centre, two-arm, parallel-group, randomized controlled trial	3 months	GCF: IL-6 TNF-α RF	Significant reduction in IL-6 and TNF in both groups (*P* < 0.05) Significantly greater reduction in group 1 compared with group 2 (*P* < 0.05)
Nguyen *et al.* (2021) [32]	*n* = 82 (1) 41 subjects with RA + PD, NSPT (2) 41 subjects with RA + PD, oral hygiene instruction	RA duration (median): (1) 3.5 (2–9) years (2) 3 (2–7) years Disease activity: (1) remission 2.4% low 7.3% moderate 53.7% high 36.6% (2) remission 4.9% low 12.2% moderate 53.6% high 29.3%	Not reported	Single-centre, two-arm, parallel-group, randomized controlled trial	6 months	DAS-28-CRP ACPA RF CRP ESR	Significant reduction in DAS-28 for group 1 6 months after NSPT (*P* = 0.013) Significant reduction of ACPA at 6 months after treatment in both arms (group 1, *P* < 0.001; group 2, *P* = 0.032) Significant reduction in ESR in group 1 (*P* < 0.001)
Ding *et al.* (2022) [33]	*n* = 89 (1) 32 subjects with RA + PD, NSPT (2) 29 subjects with PD, NSPT (3) 8 subjects with RA (4) 20 healthy controls	RA duration not reported Moderate disease activity in RA + PD arm [mean, 3.29 (1.24)]	Not reported	Single-centre, three-arm, parallel-group, non-randomized controlled trial	6 weeks	DAS-28-ESR Serum CRP ESR ACPA IL-6	Non-significant reduction in DAS-28 in group 1 Significant reduction in serum CRP and IL-6 in both groups 1 and 2 following NSPT (*P* < 0.01)

bDMARDs: biological DMARDs; csDMARDs, conventional synthetic DMARDs; DAS-28*, unclear whether DAS-28-ESR or DAS-28-CRP score calculation; GCF, gingival crevicular fluid; NSPT, non-surgical periodontal treatment; OHI, oral hygiene instruction; PD, periodontal disease; SJ, swollen joint; TJ, tender joint; VAS, visual analogue scale for pain.

### Quality assessment of included studies

Tables 3 and 4 show the quality assessment performed on the included studies. Of the randomized controlled trials, 5 of 10 studies showed high risk of bias. Of the non-randomized studies, 7 of 11 studies showed a serious to critical risk of bias.

**Table 3:** Risk of Bias In Non-randomised Studies—of Interventions (ROBINS-I)

Study [author(s), year]	Bias attributable to confounding	Bias in selection of participants into the study	Bias in classification of interventions	Bias attributable to deviation from intended interventions	Bias attributable to missing data	Bias in measurement of outcomes	Bias in selection of the reported result	Overall risk of bias judgement
Pinho *et al.* (2009) [15]	Moderate	Low	Low	Low	Critical	Serious	Moderate	Critical
Erciyas *et al.* (2012) [16]	Low	Low	Low	Low	Low	Serious	Moderate	Serious
Bıyıkoğlu *et al.* (2013) [17]	Low	Low	Low	Low	Moderate	Serious	Moderate	Serious
Roman-Torres *et al.* (2015) [19]	Moderate	Low	Low	Low	No information	Low	Moderate	Moderate
Kurgan *et al.* (2016) [20]	Low	Low	Low	Low	Low	Moderate	Moderate	Moderate
Kurgan *et al.* (2017) [21]	Moderate	Low	Low	Low	Low	Low	Moderate	Moderate
Cosgarea *et al.* (2018) [24]	Low	Low	Low	Low	Moderate	Low	Moderate	Moderate
Zhao *et al.* (2018) [25]	Moderate	Low	Low	Low	Low	Serious	Moderate	Serious
Białowas *et al.* (2019) [27]	No information	Low	Low	Low	Low	Serious	Moderate	Serious
Kaushal *et al.* (2019) [28]	Moderate	Low	Low	Low	Low	Serious	Moderate	Serious
Ding *et al.* (2022) [33]	Low	Low	Low	Low	Low	Serious	Moderate	Serious

**Table 4:** Cochrane risk of bias tool for randomized trials

Study [author(s), year]	Risk of bias arising from the randomization process	Risk of bias attributable to deviations from intended interventions	Risk of bias attributable to missing outcome data	Risk of bias in measurement of the outcome	Risk of bias in selection of the reported result	Overall risk of bias
Al-Katma *et al.* (2007) [13]	Some concerns	Low	Low	Some concerns	Low	Some concerns
Ortiz *et al.* (2009) [14]	High	Low	Low	Some concerns	Low	High
Okada *et al.* (2013) [18]	High	Low	Low	Some concerns	Low	High
Khare *et al.* (2016) [22]	High	Low	Low	Some concerns	Low	High
Serban (2017) [23]	Low	Low	Moderate	Low	Low	Moderate
Anusha *et al.* (2019) [26]	Low	Low	Low	Low	Low	Low
Monsarrat *et al.* (2019) [29]	Low	Low	Low	Some concerns	Low	Some concerns
Moura *et al.* (2020) [30]	High	Low	Low	Some concerns	Low	High
Elsadek & Farahat (2021) [31]	High	Low	Low	Some concerns	Low	High
Nguyen *et al.* (2021) [32]	Low	Low	Low	Some concerns	Low	Some concerns

### Effect of NSPT on DAS-28

DAS-28 was reported in a total of 17 studies. DAS-28 was calculated using ESR in eight studies, in two studies using CRP and one study reported both formats. Whether CRP or ESR was used was not reported in seven studies. Of the 17 studies, 9 demonstrated a statistically significant improvement in the DAS-28 after NSPT, compared with baseline. Of the 10 studies that reported on inter-group analysis, 6 studies demonstrated a statistically significant difference between the experimental and control arms.

A recent paper by Nguyen *et al.* [32], comparing 41 subjects with RA who had NSPT with 41 RA controls, found a significant and sustained reduction in DAS-28 6 months after NSPT in the intervention arm (*P* = 0.013). This represents the longest study duration to show improved DAS-28.

Moura *et al.* [30] conducted a trial with 107 subjects, which is the largest sample size found in this systematic literature review. DAS-28 was compared between 30 RA^+^PD^+^ and 30 RA^+^PD^−^ subjects. At 45 days after NSPT, the RA^+^PD^+^ group had a reduction of DAS-28 from 4.34 to 3.12, (*P* = 0.011, 95% CI). However, this study has a high risk of bias owing to the randomization process.

In an analysis of a subgroup of 22 participants who had RA, Białowas *et al.* [27] showed that 4–6 weeks after NSPT there was a decrease of DAS-28 ESR from a median of 4.32 to 3.84 (*P* = 0.04) and a decrease of DAS-28 CRP from a median of 3.26 to 2.76 (*P* = 0.002).

Bıyıkoğlu *et al.* [17], in their small study of 15 participants who had RA and PD, found 4 weeks after treatment a reduction of DAS-28 from a mean of 4.15 to 2.14 (*P* < 0.01). However, at the 3- and 6-month re-evaluation there was no significant further change.

Cosgarea *et al.* [24] failed to demonstrate an improvement in DAS-28 after treatment at 3 or 6 months. They did, however, demonstrate a positive correlation between DAS-28 and *P. gingivalis* counts at both baseline and the 3-month re-evaluation (*r* = 0.667, *P* = 0.005).

Monsarrat *et al.* [29] failed to demonstrate a statistically significant improvement in DAS-28 after treatment in their multicentre randomized controlled trial. Notably, their sample size was very small, at only 11 participants per arm, and they failed to achieve their target sample size of 20 per arm owing to early cessation of the trial, citing the reason of futility.

Khare *et al.* [22] compared 30 RA subjects with PD who had NSPT with 30 RA subjects with PD for whom treatment was withheld. At the 3-month follow-up, there was a statistically significant reduction in the treatment arm compared with the control arm (*P* = 0.002). The mean DAS-28 reduction was 1.05 (s.e.m. 0.28; *P* < 0.05), whereas in the control group there was no mean reduction.

The full results are summarized in Supplementary Table S2, available at *Rheumatology Advances in Practice* online.

### Effect of NSPT on CRP and ESR

A total of 7 studies evaluated the effect of periodontal intervention on CRP [15, 16, 18, 21, 27, 32, 33], and 10 evaluated ESR [13–16, 21, 23, 27, 29, 32, 33]. Following NSPT, six studies demonstrated a significant reduction in ESR, and four studies demonstrated a significant reduction in CRP. Despite these studies finding significant reductions in CRP/ESR, there was a high intra-group variability of CRP/ESR. Taken together, these findings suggest that serum CRP and ESR might have limited applicability as useful indicators in determining the effect of a periodontal intervention on systemic markers of inflammation in participants with RA. Supplementary Table S3, available at *Rheumatology Advances in Practice* online, summarizes the change in serum CRP and ESR values after NSPT.

### Effect of NSPT on RF

Seven studies evaluated RF levels before and after NSPT. Five studies [18, 26, 28, 32, 33] compared RF levels at baseline and after NSPT between experimental and control groups, whereas the design of two studies [17, 24] meant that only a single cohort could be included for analysis, as a before/after score. Results were inconsistent between studies and showed high variability within cohorts, demonstrated by large standard deviations. These findings suggest that, based on the limited data available, RF does not appear to be a reliable biomarker to measure the systemic response to NSPT. Supplementary Table S4, available at *Rheumatology Advances in Practice* online, summarizes the changes in RF after NSPT.

### Effect of NSPT on ACPA level

A total of six studies evaluated serum ACPA. Three studies reported a significant reduction in serum ACPA levels after NSPT.

Zhao *et al.* [25] found a reduction in ACPA at 4 weeks after NSPT, with a mean value of 102.24 (97.70) RU/ml at baseline and 57.46 (47.96) RU/ml at 4 weeks. The changes between baseline and 4-week reassessment in ACPA were also highly correlated with the changes in mean probing depth (*r* = 0.939, *P* < 0.001).

Anusha *et al.* [26] found a significant reduction in ACPA after NSPT in all study arms (*P* < 0.001). Likewise, Nguyen *et al.* [32] found a reduction in ACPA after NSPT, which was sustained at 6 months (*P* < 0.001).

Ding *et al.* [33] failed to demonstrate a statistically significant reduction in ACPA 6 weeks after NSPT. Likewise, Okada *et al.* [18] found no reduction at 8 weeks after NSPT in ACPA in the treatment group. They did, however, find that the serum ACPA level was positively correlated with *P. gingivalis* abundance from periodontal pocket samples. Supplementary Table S5, available at *Rheumatology Advances in Practice* online, summarizes the change in serum ACPA values after NSPT.

### Effect of NSPT on ancillary biomarkers

A total of 9 out of 21 studies investigated the effect of NSPT on ancillary biomarkers. Four studies investigated the effect of NSPT on serum TNF-α. Two of the studies [16, 31] found a statistically significant reduction in TNF-α after NSPT. Two studies investigated the effect of NSPT on gingival crevicular fluid MMP-8 levels. One of the studies demonstrated a statistically significant reduction in MMP-8 in RA subjects with PD after treatment at 3-month follow-up [20]. Two studies investigated the effect of NSPT on gingival crevicular fluid IL-1β levels. One of the studies found a statistically significant reduction in gingival crevicular fluid IL-1β after treatment in RA subjects [33]. Three studies [20, 31, 33] investigated the effect of NSPT on IL-6. Two of the studies demonstrated a significant reduction at the end point of 3 months [20, 33], and one study found a significant reduction in IL-6 at 6 weeks [33]. Supplementary Table S6, available at *Rheumatology Advances in Practice* online, summarizes the effect of NSPT on ancillary biomarkers.

### Effect of NSPT on swollen and tender joint counts

A total of four studies included data on the effect of NSPT on swollen and tender joint counts. Two of these four studies reported that there was a statistically significant improvement in swollen and tender joint counts after treatment.

Ortiz *et al.* [14] found a statistically significant reduction of swollen joint counts 6 weeks after NSPT in the two treatment arms (group A, subjects with RA and PD who had treatment, and group C, subjects with RA and PD who were also taking anti-TNF medication and had treatment; *P* < 0.01 in both cases). They also found a statistically significant reduction in tender joint count in group C (*P* < 0.05) but not in group A.

Białowas *et al.* [27] found a statistically significant reduction in swollen joint count (*P* = 0.01) and tender joint count (*P* = 0.04) 6 weeks after NSPT. Neither Al-Katma *et al.* [13] nor Okada *et al.* [18] found a significant reduction in swollen or tender joint counts in their studies.

### Effect of NSPT on patient-reported outcomes

Two studies evaluated the duration of early morning stiffness [13, 27]. Neither of these studies found a significant reduction in the duration of early morning stiffness after NSPT. Two studies [15, 27] evaluated the effect of NSPT on the HAQ. Neither study demonstrated a significant improvement in HAQ after NSPT.

### Adverse events

There were no adverse events reported in any of the included studies from NSPT in the study populations. Of interest, however, in the study by Monsarrat *et al.* [29], two participants from the treatment group dropped out owing to the concerns that NSPT might trigger an RA flare.

## Discussion

This systematic review has identified and included new data providing an important update on this topic. The relationship between periodontal disease and RA, and the potential impact of NSPT on RA continue to be research areas of great interest. This is reflected in the markedly increased frequency of studies exploring these links in the last 5 years [34]. We found a total of 21 eligible articles examining the impact of NSPT on RA outcomes in participants with a diagnosis of RA. Most studies used DAS-28 as the primary outcome for assessing the impact of periodontal therapy on RA. Importantly, the majority of studies found a statistically significant reduction in DAS-28 after NSPT, supporting the existing body of literature and suggesting a direct link between periodontal disease and RA.

Study design was highly variable, with a moderate to critical risk of bias in most studies. The most common risks for biases were in measurements of outcomes and selection of the reported result for non-randomized study designs assessed using the ROBINS-I tool. For randomized controlled trials, the reporting/design of the randomization process and measurement of outcomes were most frequently areas of concern. Several trials were multiple-arm studies with a range of interventions, disease status (periodontal disease positive/negative, RA positive/negative arms and healthy controls), making meaningful inter-group comparisons difficult. Such designs also meant that there was no true control group in many cases (i.e. RA subjects with periodontal disease who did not receive a periodontal intervention).

Although systematic reviews with meta-analyses have been published examining the impact of periodontal therapy on RA [7–9], we felt that heterogeneity in study design and populations precluded quantitative data synthesis. Furthermore, our review presents up-to-date data on an expanding topic that garners great interest.

Case definitions for both periodontal disease and RA varied significantly between studies. This is important because the severity of periodontal disease and subsequent levels of oral mucosal inflammation might be an important prognostic factors. Levels of baseline RA-related systemic inflammation might have similar importance regarding RA-related outcome measures. Future studies should follow the 2017 World Workshop on the Classification of Periodontal and Peri-Implant Diseases and Conditions [35] to characterize the periodontal disease status of participants. RA case definitions should follow 2010 ACR/EULAR classification criteria [36]. In addition to differences in case definitions for both conditions, the reporting quality for clinical, demographic and socioeconomic factors was variable. Some studies provided only baseline details for the entire study cohort, rather than individual groups, making assessment of potential confounders impossible. Key RA parameters, such as disease duration, ACPA antibody status, treatment modalities and co-morbid conditions, were reported infrequently. Periodontal parameters were more consistent, but in some studies the plaque/gingival bleeding indices, which are key measures of participant compliance with treatment, were absent. However, most studies reported bleeding on probing and probing pocket depth, which are considered the key indicators of periodontal disease progression/activity [37].

Description of the periodontal interventions frequently lacked sufficient detail, with the experience of the operator (general dental practitioner, dental care professional or periodontal specialist), instruments/equipment used, thresholds for plaque control before treatment and any time limits rarely reported. In the five studies that followed subjects up to 6 months, only a single course of NSPT was provided, whereas the accepted standard of care is for ∼3-monthly supportive therapy [37]. This might explain the failure to maintain statistically significant reductions in RA biomarkers at the 6-month follow-up in two of the studies [15, 24].

Most of the included studies (16 of 21) had a primary endpoint of 3 months or less. Although this may be adequate to demonstrate proof of concept, because both RA and periodontal disease are chronic conditions, the clinical significance of acute changes after a single course of treatment is questionable. Whether the improvements seen in most studies would be sustained with continued treatment or supportive therapy remains to be investigated.

Most studies measured several RA-specific and ancillary pro-inflammatory biomarkers (e.g. RF, ACP; and TNF-α, IL-1β etc., respectively). Although identification of candidate biomarkers is an important objective, these markers were highly variable both at baseline and after treatment and did not appear to be suitable as independent measures of disease activity. Although levels of inflammatory markers offer one explanation for a common mechanism of action for the impact of periodontal therapy on both periodontal disease and RA activity, most of these biomarkers do not appear to be useful proxies for more robust clinical measures, such as DAS-28 for RA or bleeding on probing/probing pocket depth for periodontal disease. One promising biomarker as an outcome measure for assessing the impact of NSPT on RA disease activity is ACPA, for which three studies found there to be a significant reduction after treatment [25, 26, 32].

Regarding interpretation of DAS-28 reduction, future studies should take care to report consistent DAS-28 formats. This review found inconsistency in the use of either DAS-28-ESR or DAS-28-CRP, and occasionally, the format was not reported. This is particularly important because CRP has been shown to be elevated in individuals with periodontal disease and no systemic disease [38]. Therefore, there is the possibility of inflated DAS-28-CRP scores in RA patients with periodontal disease. Interestingly, periodontal treatment results in a modest reduction of serum CRP levels in healthy individuals [38]. Four studies reported on tender and swollen joint counts, but these assessments have been reported to be subjective and have limited reproducibility [39]. Future studies should consider more objective measures of synovitis, such as power Doppler sonography.

Reporting of additional outcomes was limited for most studies. Adverse events were rarely reported, although in the studies that did report them these were rare, predictable and known consequences of periodontal therapy (e.g. dentine hypersensitivity). Only two studies [16, 27] reported quality of life/patient-reported outcomes, both of which used HAQ. No studies undertook any health economic evaluations, and no studies measured patient-reported experience measures or the acceptability of treatment to patients.

This systematic review had several strengths. New data have been captured by our search, thus providing an important update on this topic. We specified open inclusion criteria to include all potentially relevant studies, including subgroups of individual study arms where relevant. We used a comprehensive risk of bias/quality assessment for all study designs. We elected to avoid meta-analyses owing to the heterogeneity of study design, high risk of bias present in most studies and clinical variability between study samples. Based on the limitations of currently available studies, we have suggested considerations for the design of future research to evaluate the impact of NSPT on RA (Box 1). A quality assessment of our review using the AMSTAR-2 tool [40] demonstrated a rating of moderate confidence in the results. Owing to NHS funding restraints, a limitation of our review was the inclusion only of research published in English. Future reviews would benefit from including non-English language studies, increasing the data available to analyse and reducing potential selection bias.

Box 1. We make these recommendations for future research

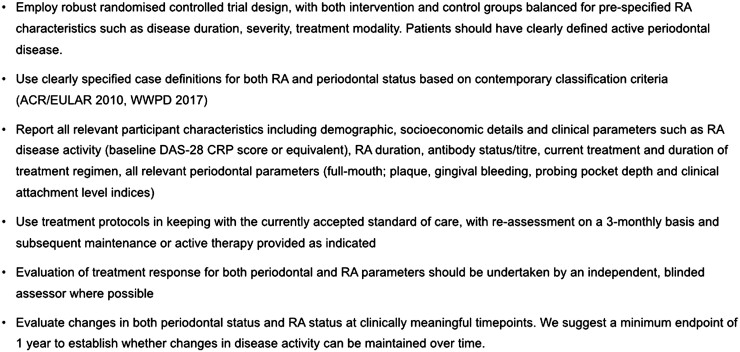

When reported, the duration of RA in the study populations was at least several years. The participants also had persistent active disease, despite pharmacotherapy. Despite such recalcitrant disease characteristics, most studies demonstrated a clinically meaningful reduction in disease activity, underscoring the potential for management of periodontal disease as a potential adjunctive measure. One study that recruited refractory RA participants but did not meet the inclusion criteria was the study by Möller *et al.* [41]. They collected data on the effect of NSPT on DAS-28-ESR. Five of eight participants had an improvement of ≥0.6 DAS-28 points at 3 months. Furthermore, they found a greater periodontal improvement in these five subjects.

Although more research is needed, we found clear support for the use of NSPT in people who have both PD and RA. Recent studies have shown that there is a higher incidence of periodontal disease in ACPA^+^ individuals who have not yet progressed to RA compared with controls [6]. We recommend that there should be a heightened awareness among dentists, both in the community and in hospitals, of this potential co-morbidity, which, if left untreated, could lead to dental infections and premature tooth loss.

It has been suggested that periodontal inflammation might precede joint inflammation and therefore that the periodontium could be the site of RA disease initiation in some individuals. Early detection/treatment of PD in the pre-RA phase could therefore delay/prevent RA [2]. Recent trials have studied individuals with established RA, with most study populations having had RA for many years before investigation. It is not known which RA population stands to benefit the most from periodontal intervention. This review demonstrates that NSPT improves disease activity in individuals with RA; however, we hypothesize that the greatest therapeutic benefit might be for those who do not yet have RA or have very early disease. A well-designed randomized controlled trial is now imperative to ascertain which population in the RA continuum stands to benefit the most. Further translational research is also required to unravel the mechanisms that underpin this treatment effect, with an integration of microbiological and immunological assessments alongside clinical outcomes.

*Funding:* There was no funding associated with this research.

*Disclosure statement:* The authors have declared no conflicts of interest.

*Registration:* The systematic review was not registered. There was no deviation from the protocol throughout the review.

## Supplementary Material

rkac061_Supplementary_Data

## Data Availability

The data used for this paper are available online through Medline/Pubmed, Embase and Cochrane Central Register.
