# Supplementary material for: Does periodontal treatment improve rheumatoid arthritis disease activity? A systematic review
Source: Rheumatol Adv Pract. 2022 Aug 17;6(2):rkac061. doi: 10.1093/rap/rkac061 (PMC9390064; doi:10.1093/rap/rkac061)
Supplement: rkac061_Supplementary_Data [file rkac061_supplementary_data.docx]

**Supplementary Material**

**Supplementary Data S1: Search Terms**

The following key terms were used:

Periodontitis OR "Periodontal disease*" OR "Aggressive Periodontitis" OR "Chronic Periodontitis" OR "Acute Nonsuppurative Periodontitis" OR "Apical Periodontitis" OR "Early-Onset Periodontitis" OR Pericementitis OR Gingivitis OR "Periodontal Abscess" OR Periodontosis OR paradontitis, "Rheumatoid Arthritis" OR "Caplan Syndrome" OR "Felty Syndrome" OR "Familial Felty's Syndrome" OR "Rheumatoid Nodule" OR "Rheumatoid Nodulosis" OR "Rheumatoid Vasculitis" OR "Sjogren Syndrome" OR "Sicca Syndrome" OR "Rheumatoid Factor*" OR "Rheumatoid Arthritis Factor*" OR "Onset Still disease" OR "Rheumatoid Disease*" OR "Chronic Rheumatoid Arthritis" OR "Inflammatory Arthritis" OR "Arthrosis Deformans" OR "Beauvais Disease" OR "chronic Progressive Polyarthritis" OR Rheumarthritis OR "Articular Rheumatism”, Non-surg* OR non-operat* OR Therap* OR Treatment OR treated orIntervention* OR "root planing" OR "root scaling" OR "dental scaling" OR "subgingival scaling" OR "supragingival scaling" OR "periodontal debridement" OR "dental debridement" OR "root surface debridement" OR "dental curettage" OR "subgingival curettage" OR "dental surg*" OR "oral surg*" OR "periodontal procedure*"

**Supplementary Table S1. Excluded studies**

| **Author (year)** | **Reason for exclusion** |
| --- | --- |
| Ribeiro (2005) | No relevant RA outcome data |
| Ranade (2012) | No relevant RA outcome data |
| Chou (2015) | No relevant RA outcome data |
| Yuce (2017) | No relevant RA outcome data |
| Yang (2017) | No RA study population |
| Artabashi-Moghadam (2018) | Unclear methodology |
| Mukherjee (2018) | Case report |
| Hashimoto (2018) | No periodontal intervention |
| Kaneko (2018) | Insufficient periodontal treatment |
| Heredia (2019) | No periodontal intervention |
| Moller (2019) | Data non-extractable |
| Marotte (2019) | No periodontal intervention |
| Chen (2019) | No relevant RA outcome data |
| Mariette (2019) | Insufficient periodontal treatment |
| Marotte (2020) | No periodontal intervention |
| Pandya (2020) | Unclear follow up of study population |
| Buwembo (2020) | No relevant RA outcome data |
| Akbari (2021) | No inclusion/exclusion criteria  Unclear methodology |
| Martu (2021) | No relevant RA outcome measures |

**Supplementary Table S2. Summary of changes in DAS-28 scores following periodontal treatment**

| Author (year) | Study duration | **Intra-group** reduction in DAS28 in **control** arm (mean, SD, p-value where given) | **Intra-group** reduction in DAS28 after periodontal treatment in **experimental** arm (mean, SD, p-value where given) | **Inter-group** significance level |
| --- | --- | --- | --- | --- |
| Al-Katma (2007) | 8 weeks | -0.5 (0.47) | 0.6 (0.5) p<0.05 | P<0.05 |
| Ortiz (2009) | 6 weeks | RA + PD, no treatment 0.31 (0.36) | RA + PD + supra/sub-gingival scaling 1.58 (0.46) p<0.01 | 4-group comparison p=0.0269  Treatment vs no treatment p=0.005 |
|  | 6 weeks | RA + PD, no treatment but taking anti-TNF medication  0.24 (0.55) | RA + PD + supra/sub-gingival scaling, and taking anti-TNF medication   1.42 (0.46) p<0.05 |  |
| Pinho (2009) | 6 months | Missing data for baseline control arm | 0.65 (0.37) | 3 months p=0.0381  6 months p=0.5755 |
| Erciyas (2012) | 12 weeks | n/a | Moderate/high disease activity group:  2.31 (0.21) p<0.001 | p=<0.001 |
|  | 12 weeks |  | Low disease activity group:  0.24 (0.12) p<0.001 |  |
| Okada (2013) | 8 weeks | 0.01 (0.04) p=0.95 | 0.37 (0.04) p=0.02 | p=0.02 |
| Biyikoglu (2013) | 1 month | n/a | 2.01 (0.31) p<0.01 | n/a |
|  | 6 months |  | 2.06 (0.32) p>0.05 | n/a |
| Kurgan (2016) | 12 weeks | -0.4 (median score) | -0.5 (median score) | p=0.580 |
| Kurgan (2017) | 12 weeks | n/a | -0.5 (0.48), p=0.307 | n/a |
| Khare (2016) | 12 weeks | -0.05 (0.22) | 1.05 (0.28) p<0.05 | p=0.002 |
| Serban (2017) | 6 months | 0.2 (0.24) | 0.4 (0.44) | P=0.112 |
| Cosgarea (2018) | 4 weeks | n/a | 0.1 (median) p>0.05 | n/a |
| Zhao (2018) | 4 weeks | 0.22 (0.35) | 1.15 (0.33) p<0.001 | Not reported |
| Bialowas (2019) | 6 weeks | n/a | DAS28-ESR 0.48 (median) p=0.04  DAS28-CRP 0.5 (median) p=0.002 | n/a |
| Monsarrat (2019) | 6 weeks | 0.35 (0.32) | 0.28 (0.46) p>0.05 | p= 0.22 |
| Moura (2020) | 45 days | 0.04 (0.37) | 1.34 (0.21) p=0.011 | Not reported |
| Nguyen (2021) | 6 months | 0.5 (median score) | 1.0 (median score) p<0.001 | P=0.013 |
| Ding (2022) | 6 weeks | n/a | 0.67 (0.45) p=0.06 | n/a |

A summary of the changes in the DAS-28 scores following periodontal treatment. The changes are given between baseline and re-assessment (intra-group) for both the control and the experimental arms. The statistical significance level between the trial arms (inter-group) is given where reported.

RA = rheumatoid arthritis; PD = periodontal disease; CRP = C-reactive protein; TNF = tumour necrosis factor - alpha

**Supplementary Table S3. Change in serum CRP and ESR levels**

| **Author (year)** | **Change in CRP Control arm (mean, S.D.)** | **Change in CRP Experimental arm (mean, S.D.)** | **Change in ESR Control arm (mean, S.D.)** | **Change in ESR Experimental arm (mean difference, S.D.)** | **Time of evaluation** | **Comment** |
| --- | --- | --- | --- | --- | --- | --- |
| Al-Katma 2007 | NR | NR | -8.1 (8.41) | 9.9 (5.89) | 8 weeks | Experimental arm showed statistically significant improvement in ESR vs. control arm (P<0.05) |
| Ortiz 2009 | NR | NR | 5.5 (9.91)* | 42 (8.53)* | 8 weeks | DMARDs only  No statistisically significant difference in ESR between experimental and control groups (P=0.064) |
|  | NR | NR | 10 (5.93)* | 29 (7.54)* |  | DMARDs + anti TNF-a  Statistically significant difference in ESR between experimental and control groups (P<0.05) |
| Pinho 2009 | 0.06 (0.07) | 0.04 (0.40) | 6.47 (5.16) | 2.13 (4.79) | 6 months | No statistically significant differences between groups |
| Erciyas 2012 | 9 | 0.3 | 19.53 (5.11) | 2.9 (2.21) | 3 months | CRP and ESR decreased in both Low (P < 0.05) and Moderate-High (P < 0.001) disease activity groups following treatment |
| Okada 2013 | 0.05 (0.02) | 0.02 (0.03) | NR | NR | 8 weeks | No significant difference between groups (P=0.86) |
| Kurgan 2016 | NR | 1.65 (2.09) | NR | -3.82 (7.66) | 3 months | Subgroup analysis of single arm only  No statistically significant difference following treatment |
| Kurgan 2017 | NA | 0.99 (2.25) | NA | -1.53 (5.57) | 3 months | No statistically significant difference following treatment |
| Serban 2017 | NR | NR | 4 (3.27) | 2 (2.31) | 6 months | No statistically significant difference in ESR following treatment |
| Bialowas 2019 | NA | -0.11 | NA | -5 (Median) | 6 weeks | Subgroup analysis of single arm only  No statistically significant difference following treatment |
| Monserrat 2019 | NR | NR | -0.9 (6.21) | -2.27 (7.10) | 3 months | No statistically significant difference following treatment |
| Anusha 2019 | 2.52 (0.99) | Experimental arm 1:  6.33 (0.82) | 5.85 (1.87) | Experimental arm 1:  9.4 (2.16) | 6 weeks | For both ESR and CRP, statistically significant difference for all groups following treatment (P<0.0001) |
|  |  | Experimental arm 2:  6.67 (1.05) |  | Experimental arm 2: 11.87 (1.58) |  |  |
| Nguyen 2021 | -1 (1.1) | 2.3 (0.8) | 10 (5.5) | 9.5 (6.7) | 6 months | For both ESR and CRP; significant intra-group reduction of ESR in experimental arm. Non-significant inter-group analysis. |
| Ding  2022 | 8.20 (3.75) | 12.46 (1.27) | 21.17 (6.87) | 48.60 (15.88) | 6 weeks | For both ESR and CRP; significant reduction in both arms. Significant inter-group analysis p<0.01 |

Negative values represent worsening scores

*S.D. estimated as range/4

**Supplementary Table S4. Change in serum Rheumatoid Factor levels**

| **Author (year)** | **Change in RF Control arm (mean, S.D.)** | **Change in RF Experimental arm (mean, S.D.)** | **Time of evaluation** | **Comment** |
| --- | --- | --- | --- | --- |
| Okada 2013 | -13.7 (6.86) | -3.4 (4.21) | 8 weeks | No statistically significant difference between experimental and control groups (P=0.84) |
| Cosgarea 2017 | NA | -32 (median) | 3 months | Subgroup analysis of single arm only.  No statistically significant difference following treatment (P=0.88) |
| Biyikoglu 2013 | NA | 21 (median) | 6 months | Subgroup analysis of single arm only.  No statistically significant difference following treatment |
| Anusha 2019 | 0.54 (1.55) | Experimental arm 1:  2.15 (0.75) | 6 weeks | Statistically significant difference for all groups following treatment (P<0.0001) |
|  |  | Experimental arm 2:  3.35 (1.63) |  |  |
| Kaushal 2019 | -0.03 (1.54) | 0.08 (1.42) | 3 months | No statistically significant difference between experimental and control groups (P=0.29) |
| Nguyen 2021 | -4.8 (2.5) | 7.5 (9.9) | 6 months | Non-significant between experimental and control |
| Ding  2022 | 16 (21.2) | 85.7 (39.1) | 6 weeks | Significant inter-group difference |

Negative values represent worsening scores

*S.D. estimated as range/4

**Supplementary Table S5. Change in ACPA titres**

| **Author (year)** | **Change in ACPA control arm (mean, SD)** | **Change in ACPA experimental arm (mean, SD)** | **Time of evaluation** | **Comment** |
| --- | --- | --- | --- | --- |
| Zhao (2018) | 8.91 (14.46) | 44.78 (26.72) | 4 weeks | Statistically significant |
| Anusha (2019) | 132.41 (4.84) | Experimental arm 1: 164.80 (4.82) | 6 weeks | Statistically significant |
|  |  | Experimental arm 2: 174.26 (4.30) | 6 weeks | Statistically significant |
| Okada (2013) | -0.6 (6.79) | -13.7 (6.68) | 8 weeks | Non-significant |
| Kaushal (2019) | -0.21 (5.08) | -0.11 (4.02) | 8 weeks | Non-significant |
| Nguyen (2021) | 83.7 (41.2) | 113.3 (71.1) | 6 months | Significant intra-group reduction in both arms. Inter-group analysis non-significant |
| Ding (2022) | 8 (16.21) | 35.7 (22.34) | 6 weeks | Non-significant |

Negative values represent worsening scores

*S.D. estimated as range/4

**Supplementary Table S6. Effect of PMPR on ancillary biomarkers**

| **Author (year)** | **Biomarker(s)** | **Change following PMPR** | **Comment** |
| --- | --- | --- | --- |
| Erciyas (2013) | TNF-Alpha | Significant decrease (both low and medium/high disease activity arms) |  |
| Biyikoglu (2013) | IL1-B (GCF + serum)  TNF-Alpha | Significant decrease GCF IL-1B in both arms |  |
| Okada (2013) | MMP-3  IL-6  TNF-Alpha  IgG (P. Gingivalis)  Citrulline | Significant decrease IgG and citrulline |  |
| Kurgan (2016) | MMP-8  IL-6  PGE2 | Significant decreases in MMP-8, IL-6, PGE2 |  |
| Kurgan (2017) | T-PA PAI-2 | Significant decrease in T-PA | PAI-2 significantly lower in healthy controls compared with PD groups |
| Cosgarea (2018) | MMP-8  IL-1B  IL-10 | No significant decrease | MMP-8 and IL-1B significantly higher at baseline in RA + PD group compared with PD group. |
| Bialowas (2019) | TNF-A  MMP-3  MMP-9 | No significant decrease |  |
| Elsadek (2021) | RF  IL-6  TNF-A | Significant reduction in IL-6 and TNF-A |  |
| Ding  (2022) | IL-6 | Significant reduction in IL-6 |  |
